# Modelling the effect of demographic change and healthcare infrastructure on the patient structure in German hospitals – a longitudinal national study based on official hospital statistics

**DOI:** 10.1186/s12913-023-10056-y

**Published:** 2023-10-11

**Authors:** Olaf Schoffer, Dirk Schriefer, Andreas Werblow, Andrea Gottschalk, Peter Peschel, Linda A. Liang, Alexander Karmann, Stefanie J. Klug

**Affiliations:** 1 Center for Evidence-based Healthcare, Faculty of Medicine and University Hospital Carl Gustav Carus, TU Dresden, Fetscherstr. 74, Dresden, 01307 Germany; 2 Chair of Epidemiology, Department of Sport and Health Sciences, Technical University of Munich, Munich, Germany; 3 Center of Clinical Neuroscience, Faculty of Medicine and University Hospital Carl Gustav Carus, Dresden, Germany; 4 Health Economics Centre and Faculty of Business and Economics, TU Dresden, Dresden, Germany; 5 Institute For Medical informatics and Biometry, Faculty of Medicine Carl Gustav Carus, TU Dresden, Dresden, Germany; 6 Health Sciences and Public Health, TU Dresden, Dresden, Germany

**Keywords:** Inpatient care, Demographic change, Patient structure, Hospital infrastructure, Hospital planning

## Abstract

**Background:**

Effects of demographic change, such as declining birth rates and increasing individual life expectancy, require health system adjustments offering age- and needs-based care. In addition, healthcare factors can also influence health services demand.

**Methods:**

The official German hospital statistics database with odd-numbered years between 1995 and 2011 was analysed. This is a national comprehensive database of all general hospital inpatient services delivered. Official data from hospital statistics were linked at the district level with demographic and socio-economic data as well as population figures from the official regional statistics. Panel data regression, modelling case numbers per hospital, was performed for 13 diagnosis groups that characterised the patient structure. Socio-demographic variables included age, sex, household income, and healthcare factors included bed capacity, personnel and hospital characteristics.

**Results:**

The median number of annual treatments per hospital increased from 6 015 (5th and 95th percentile [670; 24 812]) in 1995 to 7 817 in 2011 (5th and 95th percentile [301; 33 651]). We developed models characterising the patient structure of health care in Germany, considering both socio-demographic and hospital factors. Demographic factors influenced case numbers across all major diagnosis groups. For example, the age groups 65–74 and 75 + influenced cerebrovascular disease case numbers (p < 0.001). Other important factors included human and material resources of hospitals or the household income of patients. Distinct differences between the models for the individual diagnosis groups were observed.

**Conclusions:**

Hospital planning should not only consider demographic change but also hospital infrastructure and socio-economic factors.

**Supplementary Information:**

The online version contains supplementary material available at 10.1186/s12913-023-10056-y.

## Introduction

In high-income countries like those in Europe, significant demographic changes are expected to not only impact economic and social systems but also public health, health services planning and policy-making [1]. For example, over the past two decades in Germany, the birth deficit combined with an increase in life expectancy has resulted in a sustained change in the age structure of the population [2–4]. This demographic change has led to a decrease in the proportion of children and youths, while the proportion of the older population has increased [5]. As a result, the demand for age-specific health services has changed. In the period 2005–2017, an increasing number of inpatients with cerebrovascular diseases, specific to the age group 75 + years and a decreasing number of inpatients with diseases of the appendix, specific to the ages 0–17 years were treated [6]. Although cerebrovascular diseases such as stroke are stable or declining in age-specific incidence, the absolute number of events in the older age cohorts is expected simply due to the predicted increase in persons aged 75 + years [7]. Such changes highlight the importance of appropriate health resource planning.

With regard to hospital care planning, bed capacity is an important indicator. In Germany, the bed capacity of hospitals is determined by the state hospital plans, which only include hospitals that are reimbursed for treatment costs by the health insurance funds (§ 108 SGB V). As the demand for beds is, among other factors, dependent on patient numbers, a reliable assessment of changing inpatient numbers is necessary in order to model the demand for beds.

There is consensus that the number of inpatient treatments is associated with demographic effects, with this association being taken into consideration when projecting case numbers [8–12]. In order to model inpatient case numbers, the age-specific case numbers per diagnosis group are combined with the population projection, assuming constant diagnosis-specific case numbers per 100 000 inhabitants [8, 9]. This approach, however, has its limitations. On the one hand, the dynamics relating to the utilisation and changes in patient mobility are not taken into consideration. On the other hand, hospital infrastructure and societal context, such as social structure and urbanisation, which are constantly changing and can influence the demand for health services, are ignored.

The aim of this analysis was to explore the effect of demographic change on the patient structure in German hospitals, with a particular focus on age-specific morbidity and to adjust estimates for the influence of other determinants. Suitable data were available from the official German hospital statistics combined with regional demographic and socio-economic data, which we therefore used. Thus, in the constructed models for the chosen age-specific diseases, factors such as hospital infrastructure, societal structure and urbanisation were taken into account. These estimates may provide a more precise understanding of the effects on inpatient case numbers and thus an optimised long-term planning of healthcare resources in hospitals.

## Methods

This study is part of the project “Differentiation and relevance of age-specific diagnosis groups against the background of demographic change in Saxony (ADia)”, which was funded by the Roland Ernst Foundation for Healthcare in the period 03/2012-10/2014.

### Data sources and study design

In this secondary data analysis, official data from hospital statistics for all odd-numbered years between 1995 and 2011 from the Research Data Centre (FDZ) of the Federal and State Statistical Offices were analysed. These hospital statistics refer to an annual full census of patients treated on an inpatient basis in German hospitals, as well as the material and personnel resources of these institutions. In Germany by law, every inpatient admission must be reported as a depersonalised case making this a comprehensive nationwide database of hospital services statistics. Following an application procedure, these data are available for scientific analysis on a contractual basis. The data were used by means of so-called controlled remote data processing, which was carried out by the Statistical Office of the State of Saxony in accordance with the predefined analysis syntax of the TU Dresden.

In order to investigate demographic and socio-economic factors as explanatory variables for the patient structure, the hospital statistics were linked with data from the Federal Institute for Research on Building, Urban Affairs and Spatial Development (BBSR) as well as population figures from the official regional statistics [13, 14].

Socio-demographic variables such as average household income and the population proportions per age group and gender were assigned to patients based on their place of residence. Subsequently, a catchment area comprising at least 95% of all patients, was determined for each hospital, and the socio-demographic variables were weighted and averaged across the catchment area. The proportion of patients in a residential district was applied as a weighting factor to all patients in the catchment area. It was thereby possible to depict the distribution of socio-demographic characteristics in the catchment area of each individual hospital.

The study design followed a longitudinal approach, with cohorts established at the hospital level.

### Inclusion and exclusion criteria

All general hospitals operating in Germany were included in the analysis (i.e. exclusion of purely psychiatric or neurological hospitals, day clinics, night clinics and military hospitals). In addition, only hospitals that documented inpatient treatment and had no missing values with regard to the explanatory variables (complete case analysis) were included per survey year.

### Utilised variables

In order to explain the patient structure, the case numbers per hospital for the main diagnosis groups (classified according to the German modification of the International Classification of Diseases, ICD-10-GM) between 2005 and 2010 were analysed as the dependent variable [15]. Previously, we identified 13 diagnosis groups, which best describe the inpatient services utilised among various age groups in a ranked order [16]. These indicator diagnoses accounted for one-third of all inpatient cases in Germany [17]. These indicator diagnosis groups were A00-A09 (intestinal infectious diseases), I20-I25 (ischemic heart diseases), I30-I52 (other forms of heart disease), I60-I69 (cerebrovascular diseases), J30-J39 (other diseases of the upper respiratory tract), K35-K38 (diseases of the appendix), M00-M25 (arthropathies), M40-M54 (dorsopathies), O30-O48 (maternal care related to the foetus and amniotic cavity and possible delivery problems), O60-O75 (complications of labour and delivery), O80-O82 (encounter for delivery), P05-P08 (disorders of the newborn related to length of gestation and foetal growth), and S00-S09 (injuries to the head). In order to represent all cases, the total number of inpatient cases treated in hospitals was also considered as a dependent variable.

For socio-demographic explanatory variables, the proportion of inhabitants within defined age groups (0–17 years, 18–65 years, 65–74 years and 75+), gender distribution (ratio of total male to female population), mean monthly household income per inhabitant and aggregated district type where the hospital was located as a proxy for the level of urbanisation (urban core, surrounding populated areas, surrounding rural areas or rural areas) [13] were analysed.

For hospital-specific explanatory variables, the number of beds, full-time staff equivalent (physicians, non-physician staff as well as nursing staff), the average severity of cases (simulated case mix index) [18] outsourcing (staff-material costs ratio) [19], ownership of the hospital (private, public, non-profit), as well as billing according to the Diagnosis-related Group (DRG) system, were considered. As further hospital-specific explanatory variables we computed the Herfindahl-Index [20] and the Gini coefficient [21]. The Herfindahl-Index represents the level of competition to which a hospital is exposed and was defined as

$$H = \sum\nolimits_{i = 1}^N {{{\left( {\frac{{{x_i}}}{{\sum\nolimits_{j = 1}^N {{x_j}} }}} \right)}^2}} ,$$

where $${x}_{i}$$ is the number of patients for the hospital $$i$$$$=1,\dots ,N$$. For every hospital the reference $$1,\dots ,N$$ was redefined as all hospitals within the catchment area of the specific aforementioned hospital. The Gini coefficient represents the level of specialisation of a hospital and was defined as

$$G=\frac{\sum _{i=1}^{n}\sum _{j=1}^{n}\left|{x}_{i}-{x}_{j}\right|}{2n\sum _{i=1}^{n}{x}_{i}},$$

where ${x}_{i}$ is the number of patients with a main diagnosis from group $i=1,\dots ,n$ of diagnoses in this specific hospital^1^. In addition, a linear time trend component (survey year) was integrated into the model.

### Statistical procedure

For descriptive analyses, the median, the 5th and 95th percentile describing the dispersion of the investigated variables across all hospitals, as well as the underlying number of hospitals were tabulated.

As an approach to modelling the inpatient numbers, a Cobb-Douglas production function [22] was estimated via the panel regression method [23]. In order to minimise the risk of selection bias, this method was embedded in a two-stage Heckman selection model [24]. As a dependent variable, the logarithmised diagnosis-specific case numbers *per hospital* were modelled. Metric explanatory variables that did not represent proportional values were also logarithmically transformed.

Statistics for model assessment (R²) as well as parameter estimates and significance tests (t-tests) were determined. The significance level was set at 5%.

All analyses were performed with the statistical analysis software SAS (Version 9.3, Cary, North Carolina, USA).

## Results

The years 1995, 2003, 2005 and 2011 were presented descriptively although all odd years between 1995 and 2011 were taken into consideration for modelling.

### Hospitals and treated cases

Between 1 671 (1995) and 1 532 (2011) hospitals were analysed based on the available database (Table 1). The median case numbers of inpatient treatments in these hospitals increased during this period from 6 015 (1995) to 7 817 (2011).

**Table 1:** Measures of dispersion of selected main diagnoses and years – the annual number of inpatient hospital treatments per hospital (for each diagnosis, only data from hospitals that had at least one case with a main diagnosis for the respective survey years are shown) - for an overview of all observation years, diagnosis groups and explanatory variables considered, see Supplement Tables 1 and 2

Main diagnosis (Diagnosis group)	Measures of dispersion	Year			
		1995	2003	2005	2011
**All** (not diagnosis specific)	Median (Number of cases)	6 015	7 202	6 803	7 817
	5–95 percentile-interval	[670; 24 812]	[823; 28 962]	[316; 27 261]	[301; 33 651]
	N (hospitals)	1 671	1 625	1 671	1 532
**I20-I25** Ischemic heart diseases	Median (Number of cases)	264	247	190	182
	5–95 percentile-interval	[5; 2 047]	[4; 2 315]	[4; 2 098]	[7; 2 092]
	N (hospitals)	1 459	1 435	1 373	1 215
**I60-I69** Cerebrovascular diseases	Median (Number of cases)	60	161	136	127
	5–95 percentile-interval	[4; 357]	[3; 821]	[5; 783]	[7; 976]
	N (hospitals)	1 481	1 453	1 424	1 258
**K35-K38** Diseases of the appendix	Median (Number of cases)	117	100	98	96
	5–95 percentile-interval	[8; 309]	[6; 261]	[3; 240]	[11; 232]
	N (hospitals)	1 321	1 283	1 240	1 084
**M00-M25** Arthropathy	Median (Number of cases)	146	257	267	350
	5–95 percentile-interval	[6; 1 225]	[5; 1 383]	[4; 1 377]	[6; 1 587]
	N (hospitals)	1 528	1 513	1 485	1 348
**O60-O75** Complications of labour and delivery	Median (Number of cases)	15	220	326	392
	5–95 percentile-interval	[2; 143]	[16; 807]	[60; 1 058]	[94; 1 191]
	N (hospitals)	948	951	885	743
**O80-O82** Delivery	Median (Number of cases)	459	171	94	60
	5–95 percentile-interval	[129; 1 351]	[33; 698]	[17; 359]	[13; 228]
	N (hospitals)	956	936	865	729
**S00-S09** Injury to the head	Median (Number of cases)	165	139	133	161
	5–95 percentile-interval	[3; 684]	[3; 729]	[2; 736]	[4; 921]
	N (hospitals)	1 461	1 394	1 396	1 235

The diagnosis groups: other diseases of the upper respiratory tract (J30-J39), diseases of the appendix (K35-K38) and encounter for delivery (O80-O82) showed a decrease in median case numbers in 2011 when compared to the survey year 1995. In seven other diagnosis groups (A00-A09, I60-I69, M00-M25, M40-M54, O30-O48, O60-O75 and P05-P08) increases in case numbers were observed (Table 1, Supplement Table 1). Other than disorders of the newborn related to length of gestation and foetal growth (P05-P08), these trends were accompanied by a continuous decrease in the number of hospitals.

### Description of explanatory variables

In the analysed population, the median share of children and youths (0–17 years) decreased from 19.8 to 17.0% between 1995 and 2011, while the working-age population (18–65 years) decreased from 64.4 to 62.6% (Supplement Table 2). There was a continuous increase in the median share in the oldest population groups over the years: in the age group 75 years and older from 6.4 to 9.1% and in the 65 to 75 year age group from 9.1 to 11.1%. Similarly, the household income increased from 1 223 Euro to 1 587 Euro, while the ratio of men to women remained relatively constant.

There was no continuous trend regarding median bed numbers per hospital over time, although a slight decrease in 2011, with 201 beds compared to 222 beds in 1995, was observed. The median medical personnel increased continuously from 27.2 employed full-time per hospital in 1995 to 42.7 in 2011.

Regarding the DRG billing system, 60.5% of all hospitals in 2003 and 93.5% in 2011 used this billing system. The percentage share of the hospitals that were privately owned more than doubled during the investigation period from 12.6% to 1995 to 27.6% in 2011.

### Socio-demographic effects

The modelling of the patient structure related to all considered diagnosis groups showed a strong influence of demographics, particularly age, on the diagnosis-specific case numbers (Table 2). For example, the case numbers of arthropathies (M00-M25) rose with an increase in the proportion of inhabitants in the age groups 65–74 (p < 0.001). Overall, there were distinct differences between the effects of age groups on diagnosis group case numbers. The district type only revealed a minimal effect.

**Table 2:** Modelling of selected diagnosis-specific case numbers with selected socio-demographic and hospital-specific explanatory variables (1995–2011) - for an overview of all explanatory variables and diagnosis groups considered, see Supplement Table 3

		Socio-demographic factors					Hospital-specific factors						Trend (survey year minus 1994)
Main diagnosis ^[1]^ (Diagnosis group)		Share of inhabitants 18-65 years^[2]^	Share of inhabitants 65-74 J years ^[2]^	Share of inhabitants 75 J years + ^[2]^	Gender ratio (Men/Women)	Mean household income (log)	Total beds in hospital (log)	Full-time physician staff (log)	Herfindahl-Index	Public hospital ^[3]^	Non-profit hospital ^[3]^	Billing according to DRG ^[4]^	
**All**	Parameter	**0.009**	**0.014**	**-0.025**	0.354	**-0.264**	**0.360**	**0.155**	**-0.304**	-0.003	0.008	**-0.025**	**0.023**
	p-value	**0.002**	**< 0.001**	**< 0.001**	0.230	**< 0.001**	**< 0.001**	**< 0.001**	**< 0.001**	0.776	0.533	**< 0.001**	**< 0.001**
**I20-I25**	Parameter	**-0.021**	-0.001	**-0.035**	-1.473	0.206	**0.242**	**0.269**	**0.727**	**-0.089**	-0.069	**-0.188**	**-0.019**
	p- value	**0.005**	0.888	**0.004**	0.064	0.202	**< 0.001**	**< 0.001**	**< 0.001**	**0.002**	0.056	**< 0.001**	**< 0.001**
**I60-I69**	Parameter	**-0.099**	**-0.039**	**-0.103**	0.359	**5.845**	**0.257**	-0.048	**0.709**	-0.045	-0.070	-0.019	**-0.047**
	p- value	**< 0.001**	**< 0.001**	**< 0.001**	0.699	**< 0.001**	**< 0.001**	0.150	**< 0.001**	0.171	0.089	0.354	**< 0.001**
**K35-K38**	Parameter	-0.011	**-0.062**	**-0.171**	**-3.000**	-0.149	**0.522**	**0.208**	-0.171	-0.008	0.036	**-0.069**	**0.021**
	p- value	0.111	**< 0.001**	**< 0.001**	**< 0.001**	0.303	**< 0.001**	**< 0.001**	0.082	0.764	0.281	**< 0.001**	**< 0.001**
**M00-M25**	Parameter	**0.019**	**0.046**	**0.029**	0.723	0.143	**0.526**	**0.180**	-0.180	**-0.226**	-0.058	**-0.075**	**0.010**
	p- value	**0.030**	**< 0.001**	**0.034**	0.425	0.441	**< 0.001**	**< 0.001**	0.159	**< 0.001**	0.154	**< 0.001**	**0.038**
**O60-O75**	Parameter	**-0.106**	**0.214**	**-0.217**	**9.131**	**6.097**	**0.289**	0.051	-0.120	**0.198**	**0.282**	**1.121**	0.004
	p- value	**< 0.001**	**< 0.001**	**< 0.001**	**< 0.001**	**< 0.001**	**0.037**	0.541	0.633	**0.004**	**0.004**	**< 0.001**	0.700
**O80-O82**	Parameter	**0.043**	**-0.071**	**-0.052**	0.086	**2.739**	**0.427**	**0.236**	0.294	0.008	-0.035	**-0.992**	**-0.109**
	p- value	**0.002**	**< 0.001**	**0.020**	0.952	**< 0.001**	**< 0.001**	**< 0.001**	0.122	0.873	0.636	**< 0.001**	**< 0.001**
**S00-S09**	Parameter	**0.027**	0.005	**-0.067**	-0.477	**-1.511**	**0.384**	**0.292**	**-0.212**	**-0.058**	**-0.067**	**-0.081**	**0.034**
	p- value	**< 0.001**	0.516	**< 0.001**	0.486	**< 0.001**	**< 0.001**	**< 0.001**	**0.026**	**0.018**	**0.028**	**< 0.001**	**< 0.001**

[1] For further diagnoses and explanatory variables, see Supplement Table 3, [2] Reference category: Share of inhabitants from 0–17 years, [3] Reference category: Private hospital, [4] Reference category: No billing according to DRG,

**Bold: p < 0.05**

The household income, a proxy for socio-economic indicator of affluence, showed significant parameter estimates for the majority of the diagnoses. For example, with increasing income, the case numbers for complications with delivery (O60-O75: p < 0.001) increased, while case numbers of injuries to the head (S00-S09: p < 0.001) decreased.

### Hospital-specific factors

For all diagnosis groups, the bed capacity of the hospitals was a significant explanatory variable, with number of cases increasing alongside increasing bed capacity. In addition, the impact of staffing on the case numbers was significant in most of the panel models. With higher numbers of physician staffing, more cases with head injuries (S00-S09) were treated (adjusted estimate 0.292; p < 0.001).

The competition between hospitals had varying effects on the patient structure based on specific diagnosis groups. In hospitals with a higher Herfindahl Index, fewer cases with head injuries (S00-S09) were treated on an inpatient basis compared to other hospitals (estimate − 0.212; p = 0.026), while the opposite effect was found for ischemic heart disease (estimate 0.727; p < 0.001).

An additional hospital-specific factor, billing according to DRG, showed mostly significant parameter estimates, whereby the introduction of the DRG system was predominantly associated with decreased case numbers.

Regarding hospital operations, only a few parameter estimates were significant. Compared to private hospitals, more treatments for complications with delivery (O60-O75) in non-profit (estimate 0.282; p = 0.004) and public hospitals (estimate 0.198; p = 0.004) were observed. There were significantly fewer treatments for ischemic heart disease (I20-I25) in public hospitals (estimate − 0.089; p = 0.002) and injuries to the head (S00-S09) in non-profit hospitals (estimate − 0.067; p = 0.028) compared to private hospitals.

A significant continuous trend in case numbers over the survey years was found for all diagnosis groups with the exception of arthropathies (M00-M25), mostly associated with increasing case numbers over time.

## Discussion

This study analysed changes in the patient structure based on indicator diagnoses within inpatient services in general hospitals between 1995 and 2011. In addition, the effect of various characteristics of the treating hospitals, as well as the socio-demographic effects of the population in the catchment area of these hospitals, were used to model the patient structure.

The number of general hospitals decreased by 17% between 1995 and 2011, while the number of cases treated on an inpatient basis increased [25]. This continual decrease in the number of hospitals in order to ensure the financial feasibility and efficiency of the inpatient treatment network has been described previously [26–31]. There was a heterogeneous effect in the case numbers of the indicator diagnoses. Thus, when drawing conclusions regarding the total number of case numbers, the heterogeneity with regard to the main diagnosis groups should not be ignored. Diseases of the appendix (K35-K38), which predominantly occur in the younger age groups, showed decreasing case numbers. In contrast, there was an increase in the case numbers of cerebrovascular diseases (I60-I69), which occur predominantly among older age groups, confirming an association with demographic change. However, not all effects can be attributed to demographic change, as the case numbers per hospital for injuries to the head (S00-S09), which occur more often in the younger age groups, remained constant between 1995 and 2011. It is therefore essential in models to take other explanatory variables into account, as was undertaken in the current analysis.

The changes in case numbers did not, however, occur continuously over time for all diagnosis groups. For example, in the diagnosis group: pregnancy, childbirth and puerperium (ICD O00-O99) a drastic change in case numbers occurred with the introduction of the DRG system in 2003, in accordance with the National Health Insurance (GKV) Health Reform Bill. It can therefore be assumed that the shift in the birth diagnoses from O80-O82 to O60-O75 is a coding and documentation effect brought about by the introduction of the DRG, which needs to be distinguished from other morbidity effects [32]. A significant decrease in case numbers associated with the introduction of the DRG, independent of a long-term trend, was also found for ischemic heart disease, diseases of the appendix, arthropathies and head injuries, supporting previous observations [33]. DRG documentation effects were therefore taken into consideration in the models.

The diagnosis specification needs a certain level of detail, which we provided here by the diagnosis groups. Modelling took diagnosis-specific heterogeneity into account, with a separate model estimated for each indicator diagnosis. Across all the diagnosis groups there was a strong influence of demographic and hospital-related characteristics, while the level of urbanisation only substantially influenced a few diagnosis groups. The location of the hospital only minimally influenced the patient structure.

Additionally, the demographic structure in the catchment area of the hospitals was significant for all diagnosis groups. This conforms to the observation that there is an increase in specific diseases and multimorbidity in the older population [34] and that there are sex differences regarding morbidity and mortality [35]. The influence of demographic effects has also been noted as relevant in other studies [8–11, 34].

At the same time, other context-related factors such as the demand for hospital infrastructure (planning of bed capacity) also need to be taken into consideration in addition to demographic effects, as seen in a review by Victoor et al. [36]. The review identified the importance of how patients choose healthcare services. For example, during the COVID-19 pandemic, bed capacities and distribution between departments was strongly impacted [37]. Such shifts in inpatient services can further exacerbate the unmet need or missed care of non-COVID-19 illnesses and should be considered via the integration of surveillance modelling data [38]. Nonetheless, external contextual factors such as hospital infrastructure should be considered when modelling patient structures for optimised resource planning, not only demographic effects.

Furthermore, the continuous trend with regard to survey year also showed different, but mostly significant effects between the diagnosis groups, confirming that the case number change was heterogeneous with regard to the diagnosis groups. This alludes to the conclusion that in addition to the explanatory variables analysed, other factors may also influence the diagnosis-specific case numbers, particularly medical and technological advances [39], but are difficult to quantify. Future studies should consider this point.

As for socio-demographic factors, national and international studies show that socially disadvantaged persons do not benefit significantly from the improved health conditions of the entire population [40–45]. The influence of social status, represented here by the household income was also evident in the above analysis.

A sustained difference in demographic changes between rural and urban areas has been shown previously [46–48]. While these differences were already described in the explanatory variables for demography (age proportions and gender ratios), significant effects with regard to the level of urbanisation at the hospital location on the patient structure were only identified for a few diagnosis groups. Since, after taking into account the demographic effects, only a small share of the variability was explained by the level of urbanisation, it is advisable for future modelling and hospital planning to consider all conceivable demographic effects.

An assessment of the inpatient sector for the period 2007–2012 came to a similar conclusion that the development of case numbers differs between the individual medical groups [49]. In particular, there are distinct differences between the plannable services (large increase in case numbers) and acute cases (rather limited increase in case numbers).

### Strengths and limitations

The national and comprehensive database of the official hospital statistics offers a near complete overview regarding general hospitals and their services in Germany. Due to the legal basis of these data, a low potential for bias due to non-reporting can be assumed. We analysed 9 years of hospital statistics between 1995 and 2011, covering periods before and after the introduction of the DRG in 2003, where DRG-related coding effects could have impacted the estimates [50]. In addition, we applied a broad set of variables together with the panel regression method, which allows for better estimation of variables considering cross-sectional and longitudinal effects, thus improving the patient structure estimates. Even though the observation period of the study ended in 2011 and covered only odd-numbered years due to the project duration and resource constraints, the analysis covered a long period of 17 years. This enabled conclusions to be drawn that are valid for the long term and likely remain relevant today and for the future.

A limitation was the restriction of the database to general hospitals. Conclusions about the outpatient sector and the substitution of inpatient by outpatient services could not be drawn [51]. In addition, psychiatric and neurologic diseases may have been underrepresented in this analysis.

It should be noted that hospital planning is the responsibility of the federal states. In some federal states there are specialised programs in the hospital plans that should guide the services which are rendered. Reference to individual federal states was not possible in this analysis. Nevertheless, the analysis may serve to refine the estimation of case numbers, by placing the focus on explanatory variables that have yet to be considered in hospital planning in Germany.

## Conclusions

Diagnosis-specific models offer a more accurate description of the associations and future developments regarding the patient structure of the inpatient sector in Germany. In terms of hospital resource planning, it is important that in addition to demographic effects such as time trends, changes in hospital infrastructure and differences between the various diagnosis groups are taken into consideration. With the knowledge and consideration of the relevant hospital-specific and socio-demographic tuning parameters available here, the currently planned German hospital reform [52] could be implemented in a well-targeted manner. Thus, the intentional and gradual prioritisation against the background of demographic change can and must be accentuated for certain diagnosis and age groups, while our modelling approach allows one to trace the impact of changing infrastructure and regional competition among hospitals. Future studies based on more recent hospital statistics should further enhance this process of prioritisation.

### Electronic supplementary material

Below is the link to the electronic supplementary material.

Supplementary Material 1

## Data Availability

The datasets generated and/or analysed during the current study are not publicly available. Restrictions apply to the availability of these data, which were used under license for the current study. However, the data are available from the Research Data Centre (FDZ) of the Federal and State Statistical Offices (forschungsdatenzentrum@statistik.sachsen.de) upon reasonable request.

## Footnotes

1 In the main text of the article, only selected explanatory variables are presented. For a complete overview see Supplement.

## Declarations

### Licensing committee

Use of data according to analysis plan approved by Research Data Centre (FDZ) of the Federal and State Statistical Offices. The FDZ has granted administrative permission to have access to the data.

### Guidelines and regulations

All methods were carried out in accordance with relevant guidelines and regulations.
