# Supplementary material for: Modelling the effect of demographic change and healthcare infrastructure on the patient structure in German hospitals – a longitudinal national study based on official hospital statistics
Source: BMC Health Serv Res. 2023 Oct 11;23:1081. doi: 10.1186/s12913-023-10056-y (PMC10566170; doi:10.1186/s12913-023-10056-y)
Supplement: Supplementary file 1 — Supplementary Material 1 [file 12913_2023_10056_MOESM1_ESM.docx]

**Supplement Table 1**: Measures of dispersion of the main diagnoses – annual number of inpatient hospital treatments per hospital (for each diagnosis, only data from hospitals that had at least one case with a main diagnosis for the respective survey years were shown)

| **Main diagnosis**  **(Diagnosis group)** | **Measures of dispersion** | **Year** | | | | | | | | |
| --- | --- | --- | --- | --- | --- | --- | --- | --- | --- | --- |
|  |  | **1995** | **1997** | **1999** | **2001** | **2003** | **2005** | **2007** | **2009** | **2011** |
| **All** (not diagnosis specific) | Median (Number of cases) | 6 015 | 6 135 | 6 438 | 6 534 | 7 202 | 6 803 | 7 506 | 7 640 | 7 817 |
|  | 5-95 percentile-interval | [670; 24 812] | [572; 26 130] | [521; 25 796] | [430; 26 481] | [823; 28 962] | [316; 27 261] | [290; 32 375] | [320; 32 675] | [301; 33 651] |
|  | N (hospitals) | 1 671 | 1 710 | 1 669 | 1 734 | 1 625 | 1 671 | 1 615 | 1 584 | 1 532 |
| **A00 - A09**  Intestinal infectious diseases | Median (Number of cases) | 45 | 48 | 54 | 55 | 67 | 74 | 106 | 138 | 153 |
|  | 5-95 percentile-interval | [3; 331] | [4; 351] | [4; 374] | [3; 364] | [4; 505] | [4; 503] | [4; 567] | [7; 618] | [7; 647] |
|  | N (hospitals) | 1 360 | 1 380 | 1 316 | 1 390 | 1 384 | 1 344 | 1 289 | 1 249 | 1 191 |
| **I20-I25**  Ischemic heart diseases | Median (Number of cases) | 264 | 263 | 282 | 265 | 247 | 190 | 166 | 171 | 182 |
|  | 5-95 percentile-interval | [5; 2 047] | [4; 2 159] | [5; 2 203] | [2; 2 198] | [4; 2 315] | [4; 2 098] | [4; 2 067] | [7; 2 100] | [7; 2 092] |
|  | N (hospitals) | 1 459 | 1 488 | 1 407 | 1 486 | 1 435 | 1 373 | 1 308 | 1 257 | 1 215 |
| **I30-I52**  Other forms of heart disease | Median (Number of cases) | 280 | 309 | 348 | 352 | 358 | 370 | 428 | 455 | 473 |
|  | 5-95 percentile-interval | [5; 827] | [5; 954] | [4; 1 033] | [4; 1 154] | [5; 1 227] | [6; 1 396] | [5; 1 715] | [14; 1 833] | [10; 1 910] |
|  | N (hospitals) | 1 509 | 1 525 | 1 465 | 1523 | 1 482 | 1 424 | 1 365 | 1 306 | 1 266 |
| **I60-I69**  Cerebrovascular diseases | Median (Number of cases) | 60 | 51 | 53 | 168 | 161 | 136 | 130 | 129 | 127 |
|  | 5-95 percentile-interval | [4; 357] | [3; 404] | [3; 472] | [3; 749] | [3; 821] | [5; 783] | [5; 918] | [8; 934] | [7; 976] |
|  | N (hospitals) | 1 481 | 1 492 | 1 435 | 1 510 | 1 453 | 1 424 | 1 355 | 1 310 | 1 258 |
| **J30-J39**  Other diseases of the upper respiratory tract | Median (Number of cases) | 231 | 216 | 213 | 175 | 165 | 113 | 96 | 114 | 107 |
|  | 5-95 percentile-interval | [1; 1 210] | [1; 1 123] | [1; 1 083] | [1; 1 160] | [1; 1 191] | [1; 1 110] | [1; 1 169] | [1; 1 184] | [1; 1 222] |
|  | N (hospitals) | 1 284 | 1 261 | 1 206 | 1 259 | 1 228 | 1 171 | 1 137 | 1 032 | 999 |
| **K35-K38**  Diseases of the appendix | Median (Number of cases) | 117 | 112 | 110 | 110 | 100 | 98 | 93 | 92 | 96 |
|  | 5-95 percentile-interval | [8; 309] | [3; 314] | [4; 304] | [5; 289] | [6; 261] | [3; 240] | [4; 242] | [11; 227] | [11; 232] |
|  | N (hospitals) | 1 321 | 1 340 | 1 273 | 1 303 | 1 283 | 1 240 | 1 173 | 1 120 | 1 084 |
| **M00-M25**  Arthropathy | Median (Number of cases) | 146 | 157 | 192 | 219 | 257 | 267 | 325 | 335 | 350 |
|  | 5-95 percentile-interval | [6; 1 225] | [6; 1 232] | [6; 1 271] | [4; 1 290] | [5; 1 383] | [4; 1 377] | [4; 1 488] | [5; 1 556] | [6; 1 587] |
|  | N (hospitals) | 1 528 | 1 545 | 1 495 | 1 546 | 1 513 | 1 485 | 1 428 | 1 386 | 1 348 |
| **M40-M54**  Dorsopathies | Median (Number of cases) | 57 | 57 | 64 | 78 | 90 | 105 | 173 | 197 | 226 |
|  | 5-95 percentile-interval | [6; 775] | [5; 809] | [6; 901] | [5; 967] | [8; 1 099] | [5; 1 079] | [4; 1 253] | [6; 1 301] | [8; 1 333] |
|  | N (hospitals) | 1 543 | 1 563 | 1 508 | 1 569 | 1 516 | 1 499 | 1 452 | 1 408 | 1 346 |
| **O30-O48**  Maternal care related to the foetus and amniotic cavity and possible delivery problems | Median (Number of cases) | 130 | 165 | 169 | 103 | 191 | 200 | 207 | 217 | 222 |
|  | 5-95 percentile-interval | [26; 413] | [15; 570] | [27; 579] | [21; 329] | [24; 646] | [33; 675] | [44; 752] | [47; 836] | [54; 846] |
|  | N (hospitals) | 997 | 1 013 | 960 | 960 | 956 | 889 | 805 | 783 | 741 |
| **O60-O75**  Complications of labour and delivery | Median (Number of cases) | 15 | 11 | 11 | 62 | 220 | 326 | 371 | 384 | 392 |
|  | 5-95 percentile-interval | [2; 143] | [1; 78] | [1; 72] | [7; 306] | [16; 807] | [60; 1 058] | [90; 1 165] | [100; 1 224] | [94; 1 191] |
|  | N (hospitals) | 948 | 955 | 908 | 962 | 951 | 885 | 806 | 782 | 743 |
| **O80-O82**  Encounter for delivery | Median (Number of cases) | 459 | 499 | 494 | 477 | 171 | 94 | 72 | 66 | 60 |
|  | 5-95 percentile-interval | [129; 1 351] | [147; 1 381] | [150; 1 275] | [145; 1 277] | [33; 698] | [17; 359] | [13; 286] | [14; 260] | [13; 228] |
|  | N (hospitals) | 956 | 974 | 928 | 947 | 936 | 865 | 792 | 771 | 729 |
| **P05-P08**  Disorders of the newborn related to length of gestation and foetal growth | Median (Number of cases) | 22 | 30 | 29 | 42 | 39 | 40 | 51 | 59 | 64 |
|  | 5-95 percentile-interval | [1; 220] | [1; 234] | [1; 244] | [1; 247] | [1; 248] | [3; 266] | [2; 321] | [3; 352] | [3; 364] |
|  | N (hospitals) | 618 | 599 | 546 | 539 | 598 | 817 | 751 | 715 | 672 |
| **S00-S09**  Injuries to the head | Median (Number of cases) | 165 | 160 | 156 | 141 | 139 | 133 | 145 | 151 | 161 |
|  | 5-95 percentile-interval | [3; 684] | [2; 666] | [2; 686] | [3; 675] | [3; 729] | [2; 736] | [3; 888] | [4; 879] | [4; 921] |
|  | N (hospitals) | 1 461 | 1 467 | 1 412 | 1 450 | 1 394 | 1 396 | 1 347 | 1 282 | 1 235 |

**Supplement Table 2**: Measures of dispersion of the explanatory variables (the socio-demographic information is assigned to the individual hospitals according to the location of the hospital / catchment area)

| **Explanatory Variables** | **Measure of Dispersion** | **Year** | | | | | | | | |
| --- | --- | --- | --- | --- | --- | --- | --- | --- | --- | --- |
|  |  | **1995** | **1997** | **1999** | **2001** | **2003** | **2005** | **2007** | **2009** | **2011** |
|  | N (Hospitals) | 1 671 | 1 710 | 1 669 | 1 734 | 1 625 | 1 671 | 1 629 | 1 615 | 1 532 |
| Share of inhabitants 0 - 18 years (%) | Median | 19.80 | 19.70 | 19.39 | 19.16 | 18.82 | 18.35 | 17.79 | 17.20 | 16.99 |
|  | 5-95 percentile-interval | [16.8; 22.6] | [17.0; 22.4] | [16.7; 22.2] | [16.2; 22.0] | [15.1; 21.6] | [14.0; 20.9] | [13.0; 20.2] | [12.8; 19.3] | [13.1; 18.9] |
| Share of inhabitants 18 - 65 years (%) | Median | 64.41 | 64.26 | 64.01 | 63.56 | 62.96 | 62.34 | 62.21 | 62.28 | 62.57 |
|  | 5-95 percentile-interval | [62.4; 67.9] | [62.0; 67.8] | [61.8; 67.9] | [61.3; 67.3] | [60.7; 66.8] | [60.1; 66.1] | [60.1; 65.8] | [60.3; 65.4] | [60.6; 65.4] |
| Share of inhabitants 65 - 75 years (%) | Median | 9.08 | 8.96 | 9.14 | 9.58 | 10.16 | 10.92 | 11.43 | 11.55 | 11.14 |
|  | 5-95 percentile-interval | [7.8; 10.7] | [7.9; 10.5] | [8.0; 10.7] | [8.5; 11.2] | [9.1; 12.0] | [9.8; 13.0] | [10.2; 13.7] | [10.3; 14.2] | [9.9; 13.7] |
| Share of inhabitants 75 years and older (%) | Median | 6.39 | 6.72 | 6.99 | 7.34 | 7.69 | 8.10 | 8.39 | 8.84 | 9.13 |
|  | 5-95 percentile-interval | [5.1; 7.7] | [5.4; 8.0] | [5.8; 8.3] | [6.2; 8.8] | [6.4; 9.2] | [6.9; 9.7] | [7.1; 10.1] | [7.5; 10.6] | [7.8; 11.1] |
| Gender ratio (Number of Men/Women) | Median | 0.95 | 0.95 | 0.95 | 0.95 | 0.96 | 0.96 | 0.96 | 0.96 | 0.96 |
|  | 5-95 percentile-interval | [0.90; 0.99] | [0.90; 0.99] | [0.90; 0.99] | [0.91; 0.99] | [0.91; 0.99] | [0.91; 0.99] | [0.91; 1.00] | [0.92; 1.00] | [0.92; 1.00] |
| Mean household income (€) | Median | 1 223.41 | 1 256.12 | 1 314.18 | 1 396.92 | 1 426.42 | 1 483.62 | 1 543.29 | 1 583.16 | 1 587.00 |
|  | 5-95 percentile-interval | [924.2; 1 462.6] | [995.5; 1 503.7] | [1 076.4; 1 567.8] | [1 139.1; 1 677.2] | [1 164.3; 1 718.1] | [1 192.0; 1 802.9] | [1 236.1; 1 863.2] | [1 298.6; 1 906.7] | [1 305.5; 1 907.4] |
| Total beds per hospital | Median | 222.00 | 211.00 | 207.00 | 203.50 | 217.00 | 203.00 | 200.00 | 200.00 | 201.00 |
|  | 5-95 percentile-interval | [50.0; 823.0] | [41.0; 833.0] | [32.0; 776.0] | [28.0; 751.0] | [39.0; 811.0] | [20.0; 769.0] | [20.0; 804.0] | [19.0; 811.0] | [18.0; 837.0] |
| Full-time physician staff per hospital | Median | 27.20 | 28.45 | 29.30 | 29.70 | 33.70 | 34.30 | 36.50 | 38.10 | 42.65 |
|  | 5-95 percentile-interval | [4.0; 176.3] | [3.7; 178.4] | [3.0; 176.8] | [3.0; 176.2] | [4.0; 201.9] | [2.0; 205.8] | [2.6; 221.5] | [2.0; 243.9] | [2.0; 281.3] |
| Full-time non-physician staff per hospital | Median | 124.00 | 116.25 | 113.20 | 112.40 | 121.80 | 112.80 | 113.80 | 116.10 | 116.30 |
|  | 5-95 percentile-interval | [25.8; 640.7] | [23.1; 626.9] | [16.5; 601.2] | [15.0; 539.7] | [20.3; 614.7] | [11.2; 570.3] | [10.1; 597.0] | [10.5; 620.7] | [10.1; 659.6] |
| Nursing staff per hospital | Median | 114.80 | 111.30 | 109.60 | 108.20 | 112.50 | 103.20 | 102.80 | 105.40 | 110.10 |
|  | 5-95 percentile-interval | [17.0; 518.2] | [14.5; 521.6] | [10.7; 494.4] | [10.0; 481.3] | [15.0; 503.5] | [7.8; 476.1] | [7.1; 500.1] | [7.0; 515.4] | [7.0; 534.5] |
| Simulated Case-Mix-Index per hospital | Median | 0.88 | 0.90 | 0.94 | 0.92 | 0.95 | 0.97 | 0.95 | 0.94 | 0.93 |
|  | 5-95 percentile-interval | [0.5; 1.8] | [0.5; 1.8] | [0.5; 1.8] | [0.5; 1.8] | [0.6; 1.7] | [0.7; 1.9] | [0.7; 1.9] | [0.7; 2.1] | [0.7; 2.0] |
| Herfindahl-Index per hospital | Median | 0.13 | 0.13 | 0.13 | 0.13 | 0.15 | 0.14 | 0.14 | 0.14 | 0.14 |
|  | 5-95 percentile-interval | [0.02; 0.42] | [0.0; 0.4] | [0.0; 0.4] | [0.0; 0.4] | [0.03; 0.45] | [0.03; 0.45] | [0.0; 0.5] | [0.0; 0.4] | [0.03; 0.45] |
| Gini-coefficient per hospital | Median | 0.82 | 0.82 | 0.83 | 0.81 | 0.81 | 0.81 | 0.82 | 0.82 | 0.82 |
|  | 5-95 percentile-interval | [0.8; 1.0] | [0.8; 1.0] | [0.8; 1.0] | [0.7; 1.0] | [0.7; 1.0] | [0.7; 1.0] | [0.7; 1.0] | [0.7; 1.0] | [0.7; 1.0] |
| Staff-material cost ratio per hospital | Median | 2.28 | 2.31 | 2.27 | 2.16 | 2.08 | 1.91 | 1.68 | 1.59 | 1.60 |
|  | 5-95 percentile-interval | [1.4; 3.4] | [1.3; 3.5] | [1.2; 3.6] | [1.1; 3.6] | [1.2; 3.2] | [0.9; 3.3] | [0.8; 2.9] | [0.7; 2.9] | [0.7; 3.0] |
| District type:  urban core | Proportion | 29.38% | 29.59% | 30.68% | 29.12% | 28.86% | 29.80% | 29.53% | 29.97% | 31.20% |
|  | 95%-CI (Clopper-P.) | [27.24%; 31.63%] | [27.47%; 31.82%] | [28.50%; 32.95%] | [27.02%; 31.32%] | [26.70%; 31.13%] | [27.65%; 32.06%] | [27.35%; 31.81%] | [27.78%; 32.27%] | [28.92%; 33.59%] |
| District type:  Surrounding populated area | Proportion | 43.99% | 43.80% | 44.16% | 43.71% | 44.06% | 43.45% | 43.71% | 43.47% | 44.19% |
|  | 95%-CI (Clopper-P.) | [41.64%; 46.40%] | [41.48%; 46.19%] | [41.81%; 46.58%] | [41.41%; 46.09%] | [41.68%; 46.52%] | [41.10%; 45.86%] | [41.33%; 46.16%] | [41.08%; 45.93%] | [41.74%; 46.72%] |
| District type:  Surrounding rural area | Proportion | 16.16% | 16.26% | 15.46% | 17.01% | 16.68% | 16.52% | 16.27% | 15.60% | 15.14% |
|  | 95%-CI (Clopper-P.) | [14.44%; 18.01%] | [14.56%; 18.09%] | [13.77%; 17.28%] | [15.29%; 18.87%] | [14.91%; 18.58%] | [14.78%; 18.39%] | [14.53%; 18.15%] | [13.88%; 17.47%] | [13.40%; 17.04%] |
| District type:  Rural area | Proportion | 10.47% | 10.35% | 9.71% | 10.15% | 10.40% | 10.23% | 10.50% | 10.96% | 9.47% |
|  | 95%-CI (Clopper-P.) | [9.06%; 12.04%] | [8.96%; 11.89%] | [8.34%; 11.23%] | [8.78%; 11.67%] | [8.97%; 11.99%] | [8.83%; 11.79%] | [9.06%; 12.09%] | [9.49%; 12.59%] | [8.06%; 11.04%] |
| Billing according to DRG | Proportion | 0.00% | 0.00% | 0.00% | 0.00% | 60.49% | 91.32% | 93.43% | 93.19% | 93.54% |
|  | 95%-CI (Clopper-P.) | - | - | - | - | [58.14%; 62.88%] | [89.99%; 92.63%] | [92.24%; 94.59%] | [91.97%; 94.37%] | [92.32%; 94.72%] |
| Ownership of the hospital:  Non-profit | Proportion | 43.93% | 42.46% | 39.66% | 39.50% | 40.68% | 37.40% | 34.87% | 33.31% | 33.23% |
|  | 95%-CI (Clopper-P.) | [41.58%; 46.34%] | [40.15%; 44.84%] | [37.35%; 42.06%] | [37.24%; 41.85%] | [38.32%; 43.11%] | [35.12%; 39.77%] | [32.59%; 37.24%] | [31.05%; 35.67%] | [30.91%; 35.65%] |
| Ownership of the hospital:  Public | Proportion | 43.50% | 43.39% | 43.98% | 42.45% | 42.46% | 39.74% | 39.41% | 39.07% | 39.16% |
|  | 95%-CI (Clopper-P.) | [41.16%; 45.92%] | [41.08%; 45.78%] | [41.63%; 46.40%] | [40.15%; 44.81%] | [40.09%; 44.91%] | [37.43%; 42.13%] | [37.07%; 41.83%] | [36.73%; 41.50%] | [36.76%; 41.66%] |
| Ownership of the hospital:  Private | Proportion | 12.57% | 14.15% | 16.36% | 18.05% | 16.86% | 22.86% | 25.72% | 27.62% | 27.61% |
|  | 95%-CI (Clopper-P.) | [11.03%; 14.25%] | [12.55%; 15.89%] | [14.63%; 18.22%] | [16.29%; 19.94%] | [15.09%; 18.77%] | [20.89%; 24.95%] | [23.64%; 27.92%] | [25.48%; 29.87%] | [25.42%; 29.92%] |

**Supplement Table 3**: Modelling of diagnosis-specific case numbers by socio-demographic and hospital-specific explanatory variables (1995-2011)

|  | | **Socio-demographic factors** | | | | | | | | **Hospital-specific factors** | | | | | | | | | | | **Other** | | **Coefficient of determination (R²)** |
| --- | --- | --- | --- | --- | --- | --- | --- | --- | --- | --- | --- | --- | --- | --- | --- | --- | --- | --- | --- | --- | --- | --- | --- |
| **Main Diagnosis**  **(Diagnosis group)** | | **Share of inhabitants 18-65 years ^[1]^** | **Share of inhabitants 65-74 Years ^[1]^** | **Share of inhabitants 75 years + ^[1]^** | **Gender ratio (Men/Women)** | **Urban core ^[2]^** | **Surrounding populated area ^[2]^** | **Surrounding rural area ^[2]^** | **Mean household income (log)** | **Total beds in hospital (log)** | **Full-time physician staff (log)** | **Full-time non-physician staff (log)** | **Full-time nursing staff (log)** | **Median severity of cases in hospital (simulated CMI)** | **Level of Specialization (Gini-coefficient)** | **Herfindahl-Index** | **Public hospital ^[3]^** | **Non-profit hospital ^[3]^** | **Billing according to  DRG ^[4]^** | **Staff-material costs ratio (Outsourcing)** | **Intercept** | **Trend (Survey year minus 1994)** |  |
| **Total** | Parameter | **0.009** | **0.014** | **-0.025** | 0.354 | 0.044 | 0.036 | 0.015 | **-0.264** | **0.360** | **0.155** | **0.088** | **0.219** | **-0.065** | **-1.634** | **-0.304** | -0.003 | 0.008 | **-0.025** | **-0.020** | **7.408** | **0.023** | 0.982 |
|  | p-value | **0.002** | **<0.001** | **<0.001** | 0.230 | 0.501 | 0.220 | 0.540 | **<0.001** | **<0.001** | **<0.001** | **<0.001** | **<0.001** | **<0.001** | **<0.001** | **<0.001** | 0.776 | 0.533 | **<0.001** | **<0.001** | **<0.001** | **<0.001** |  |
| **A00-A09** | Parameter | **0.108** | **0.154** | **0.046** | **2.742** | 0.109 | -0.072 | -0.105 | **-1.303** | **0.333** | **0.137** | 0.037 | **0.103** | **-0.340** | **-3.748** | **0.347** | 0.020 | -0.015 | -0.002 | -0.004 | 1.576 | **0.069** | 0.890 |
|  | p-value | **<0.001** | **<0.001** | **0.001** | **0.002** | 0.548 | 0.423 | 0.142 | **<0.001** | **<0.001** | **<0.001** | 0.293 | **0.025** | **<0.001** | **<0.001** | **0.005** | 0.536 | 0.716 | 0.917 | 0.790 | 0.316 | **<0.001** |  |
| **I20-I25** | Parameter | **-0.021** | -0.001 | **-0.035** | -1.473 | -0.087 | **0.278** | **0.194** | 0.206 | **0.242** | **0.269** | **0.140** | **0.145** | **0.058** | -0.005 | **0.727** | **-0.089** | -0.069 | **-0.188** | **0.032** | **3.947** | **-0.019** | 0.935 |
|  | p-value | **0.005** | 0.888 | **0.004** | 0.064 | 0.600 | **<0.001** | **0.002** | 0.202 | **<0.001** | **<0.001** | **<0.001** | **<0.001** | **0.023** | 0.988 | **<0.001** | **0.002** | 0.056 | **<0.001** | **0.025** | **0.005** | **<0.001** |  |
| **I30-I52** | Parameter | **0.015** | **0.019** | **0.030** | 0.311 | -0.193 | 0.035 | 0.037 | -0.085 | **0.388** | 0.020 | **0.121** | **0.214** | **0.081** | **-2.514** | **0.537** | 0.021 | **0.079** | **-0.069** | **-0.060** | **2.399** | **0.026** | 0.950 |
|  | p-value | **0.012** | **0.005** | **0.001** | 0.620 | 0.145 | 0.572 | 0.450 | 0.504 | **<0.001** | 0.361 | **<0.001** | **<0.001** | **<0.001** | **<0.001** | **<0.001** | 0.354 | **0.005** | **<0.001** | **<0.001** | **0.029** | **<0.001** |  |
| **I60-I69** | Parameter | **-0.099** | **-0.039** | **-0.103** | 0.359 | **0.432** | 0.157 | 0.090 | **5.845** | **0.257** | -0.048 | **0.125** | **0.160** | 0.020 | **-8.233** | **0.709** | -0.045 | -0.070 | -0.019 | **0.040** | **-25.13** | **-0.047** | 0.885 |
|  | p-value | **<0.001** | **<0.001** | **<0.001** | 0.699 | **0.026** | 0.090 | 0.215 | **<0.001** | **<0.001** | 0.150 | **<0.001** | **0.001** | 0.493 | **<0.001** | **<0.001** | 0.171 | 0.089 | 0.354 | **0.007** | **<0.001** | **<0.001** |  |
| **J30-J39** | Parameter | 0.015 | **0.084** | **-0.135** | **8.131** | 0.009 | **0.407** | **0.267** | -0.294 | **0.780** | 0.089 | 0.013 | **0.280** | -0.122 | **-7.253** | -0.124 | -0.054 | **-0.284** | **-0.172** | 0.014 | -1.580 | **-0.030** | 0.890 |
|  | p-value | 0.332 | **<0.001** | **<0.001** | **<0.001** | 0.977 | **0.009** | **0.036** | 0.365 | **<0.001** | 0.140 | 0.852 | **0.001** | 0.053 | **<0.001** | 0.578 | 0.341 | **<0.001** | **<0.001** | 0.663 | 0.589 | **0.001** |  |
| **K35-K38** | Parameter | -0.011 | **-0.062** | **-0.171** | **-3.000** | -0.009 | 0.104 | **0.169** | -0.149 | **0.522** | **0.208** | **0.171** | 0.033 | 0.006 | **-3.668** | -0.171 | -0.008 | 0.036 | **-0.069** | **-0.038** | **7.929** | **0.021** | 0.896 |
|  | p-value | 0.111 | **<0.001** | **<0.001** | **<0.001** | 0.948 | 0.141 | **0.003** | 0.303 | **<0.001** | **<0.001** | **<0.001** | 0.385 | 0.857 | **<0.001** | 0.082 | 0.764 | 0.281 | **<0.001** | **0.005** | **<0.001** | **<0.001** |  |
| **M00-M25** | Parameter | **0.019** | **0.046** | **0.029** | 0.723 | 0.061 | 0.054 | -0.136 | 0.143 | **0.526** | **0.180** | **0.426** | 0.052 | **0.056** | **-3.788** | -0.180 | **-0.226** | -0.058 | **-0.075** | **-0.196** | -1.417 | **0.010** | 0.904 |
|  | p-value | **0.030** | **<0.001** | **0.034** | 0.425 | 0.758 | 0.558 | 0.064 | 0.441 | **<0.001** | **<0.001** | **<0.001** | 0.220 | **0.036** | **<0.001** | 0.159 | **<0.001** | 0.154 | **<0.001** | **<0.001** | 0.376 | **0.038** |  |
| **M40-M54** | Parameter | **0.071** | **0.062** | **0.061** | **-4.472** | -0.056 | -0.092 | 0.039 | **-1.291** | **0.692** | **0.281** | 0.048 | **-0.151** | -0.050 | **-3.959** | -0.131 | **-0.154** | **-0.131** | -0.020 | **-0.026** | **10.928** | **0.075** | 0.899 |
|  | p-value | **<0.001** | **<0.001** | **<0.001** | **<0.001** | 0.769 | 0.301 | 0.585 | **<0.001** | **<0.001** | **<0.001** | 0.118 | **<0.001** | 0.051 | **<0.001** | 0.293 | **<0.001** | **0.001** | 0.306 | **<0.001** | **<0.001** | **<0.001** |  |
| **O30-O48** | Parameter | **0.085** | **0.153** | **-0.048** | **6.870** | -0.121 | -0.177 | -0.156 | **-3.392** | **0.290** | **0.303** | **-0.148** | **0.442** | **0.337** | **1.686** | 0.101 | **0.092** | -0.004 | **0.314** | **0.187** | **7.945** | **0.050** | 0.776 |
|  | p-value | **<0.001** | **<0.001** | **0.010** | **<0.001** | 0.604 | 0.100 | 0.066 | **<0.001** | **0.001** | **<0.001** | **0.010** | **<0.001** | **<0.001** | **0.017** | 0.519 | **0.029** | 0.946 | **<0.001** | **<0.001** | **<0.001** | **<0.001** |  |
| **O60-O75** | Parameter | **-0.106** | **0.214** | **-0.217** | **9.131** | 0.137 | -0.060 | -0.088 | **6.097** | **0.289** | 0.051 | 0.104 | -0.142 | **0.391** | **-14.93** | -0.120 | **0.198** | **0.282** | **1.121** | 0.076 | **-33.36** | 0.004 | 0.805 |
|  | p-value | **<0.001** | **<0.001** | **<0.001** | **<0.001** | 0.712 | 0.727 | 0.519 | **<0.001** | **0.037** | 0.541 | 0.265 | 0.227 | **0.004** | **<0.001** | 0.633 | **0.004** | **0.004** | **<0.001** | 0.062 | **<0.001** | 0.700 |  |
| **O80-O82** | Parameter | **0.043** | **-0.071** | **-0.052** | 0.086 | -0.316 | -0.184 | -0.106 | **2.739** | **0.427** | **0.236** | **-0.206** | 0.134 | **-0.786** | **-2.790** | 0.294 | 0.008 | -0.035 | **-0.992** | **0.101** | **-14.30** | **-0.109** | 0.770 |
|  | p-value | **0.002** | **<0.001** | **0.020** | 0.952 | 0.260 | 0.157 | 0.295 | **<0.001** | **<0.001** | **<0.001** | **0.003** | 0.133 | **<0.001** | **0.001** | 0.122 | 0.873 | 0.636 | **<0.001** | **0.001** | **<0.001** | **<0.001** |  |
| **P05-P08** | Parameter | **-0.111** | 0.000 | **-0.087** | 0.367 | 0.284 | 0.305 | -0.076 | **-2.105** | **1.043** | **-0.198** | -0.008 | **-0.525** | **0.972** | **-3.202** | **-0.894** | -0.028 | **0.338** | 0.023 | **-0.191** | **25.881** | **0.078** | 0.785 |
|  | p-value | **<0.001** | 0.995 | **0.007** | 0.860 | 0.444 | 0.127 | 0.618 | **<0.001** | **<0.001** | **0.044** | 0.941 | **<0.001** | **<0.001** | **0.030** | **0.001** | 0.714 | **0.002** | 0.710 | **<0.001** | **<0.001** | **<0.001** |  |
| **S00-S09** | Parameter | **0.027** | 0.005 | **-0.067** | -0.477 | -0.169 | 0.050 | -0.057 | **-1.511** | **0.384** | **0.292** | **0.167** | 0.021 | **-0.064** | **-6.131** | **-0.212** | **-0.058** | **-0.067** | **-0.081** | **-0.032** | **15.301** | **0.034** | 0.944 |
|  | p-value | **<0.001** | 0.516 | **<0.001** | 0.486 | 0.238 | 0.462 | 0.296 | **<0.001** | **<0.001** | **<0.001** | **<0.001** | 0.543 | **0.003** | **<0.001** | **0.026** | **0.018** | **0.028** | **<0.001** | **0.005** | **<0.001** | **<0.001** |  |

[1] Reference category: Share of inhabitants from 0-17 years, [2] Reference category: rural area, [3] Reference category: Private hospital, [4] Reference category: no billing according to DRG, **Bold: p < 0.05**
